# High-quality genome sequence and description of *Bacillus dielmoensis* strain FF4^T^ sp. nov.

**DOI:** 10.1186/s40793-015-0019-8

**Published:** 2015-07-22

**Authors:** Cheikh Ibrahima Lo, Roshan Padhmanabhan, Oleg Mediannikov, Jérôme Terras, Catherine Robert, Ngor Faye, Didier Raoult, Pierre-Edouard Fournier, Florence Fenollar

**Affiliations:** Aix-Marseille Université, URMITE, UM63, CNRS 7278, IRD 198, Inserm U1095, Faculté de médecine, 27 Boulevard Jean Moulin, 13385 Marseille cedex 05, France; Campus International UCAD-IRD, Dakar, Senegal; Université Cheikh Anta Diop de Dakar, Laboratoire de Parasitologie générale, Fann, Senegal; Special Infectious Agents Unit, King Fahd Medical Research Center, King Abdulaziz University, Jeddah, Saudi Arabia

**Keywords:** *Bacillus dielmoensis*, Genome, Taxonogenomics, Culturomics

## Abstract

Strain FF4^T^ was isolated from the skin flora of a 16-year-old healthy Senegalese female. This strain exhibited a 16S rRNA sequence similarity of 97.5 % with *Bacillus fumarioli,* the phylogenetically closest species with standing in nomenclature and a poor MALDI-TOF-MS score (1.1 to 1.3) that does not allow any identification. Using a polyphasic study consisting of phenotypic and genomic analyses, strain FF4^T^ was Gram-positive, aerobic, rod-shaped, and exhibited a genome of 4,563,381 bp (1 chromosome but no plasmid) with a G + C content of 40.8 % that coded 4,308 protein-coding and 157 RNA genes (including 5 rRNA operons). On the basis of these data, we propose the creation of *Bacillus dielmoensis* sp. nov.

**Electronic supplementary material:**

The online version of this article (doi:10.1186/s40793-015-0019-8) contains supplementary material, which is available to authorized users.

## Introduction

The genus *Bacillus* (Cohn 1872) was created about 142 years ago [1]. Currently, the genus *Bacillus* comprised 281 species and 7 subspecies with validly published names [2]. Members of the genus *Bacillus* are environmental bacteria isolated most often from soil, food, fresh and sea water. Furthermore, they live rarely in human and animals in which they are either pathogens, such as *B. anthracis* (the causative agent of anthrax) [3, 4] and *B. cereus* (associated mainly with food poisoning) [4, 5], or saprophytes [4, 6]. Many species of the genus *Bacillus* are also isolated from different plants in which they are endophytes [7].

Recently, high throughput genome sequencing and mass spectrometric (MALDI-TOF MS) analyses of bacteria have given unprecedented access to an abundance of genetic and proteomic information [8–10]. Thus, a polyphasic approach is currently proposed to describe new bacterial taxa that includes their genome sequence, MALDI-TOF MS spectrum and major phenotypic characteristics such as Gram staining, culture, metabolic characteristics, habitat and if applicable, pathogenicity [9–11].

*Bacillus**dielmoensis* strain FF4 (= CSUR P3026 = DSM 27844) is designated as the type strain of *B. dielmoensis.* This bacterium is a Gram-positive, non-spore-forming, aerobic and motile bacillus*.* This bacterium was isolated from the skin of a healthy Senegalese female as part of a “culturomics” study aiming at cultivating bacterial species from the skin flora [12]. Here, we present a summary classification and a set of features for *B. dielmoensis* sp. nov. strain FF4^T^ together with the description of the complete genomic sequencing and annotation. These characteristics support the circumscription of the species *B. dielmoensis.*

## Organism information

### Classification and features

**Fig. 1:**
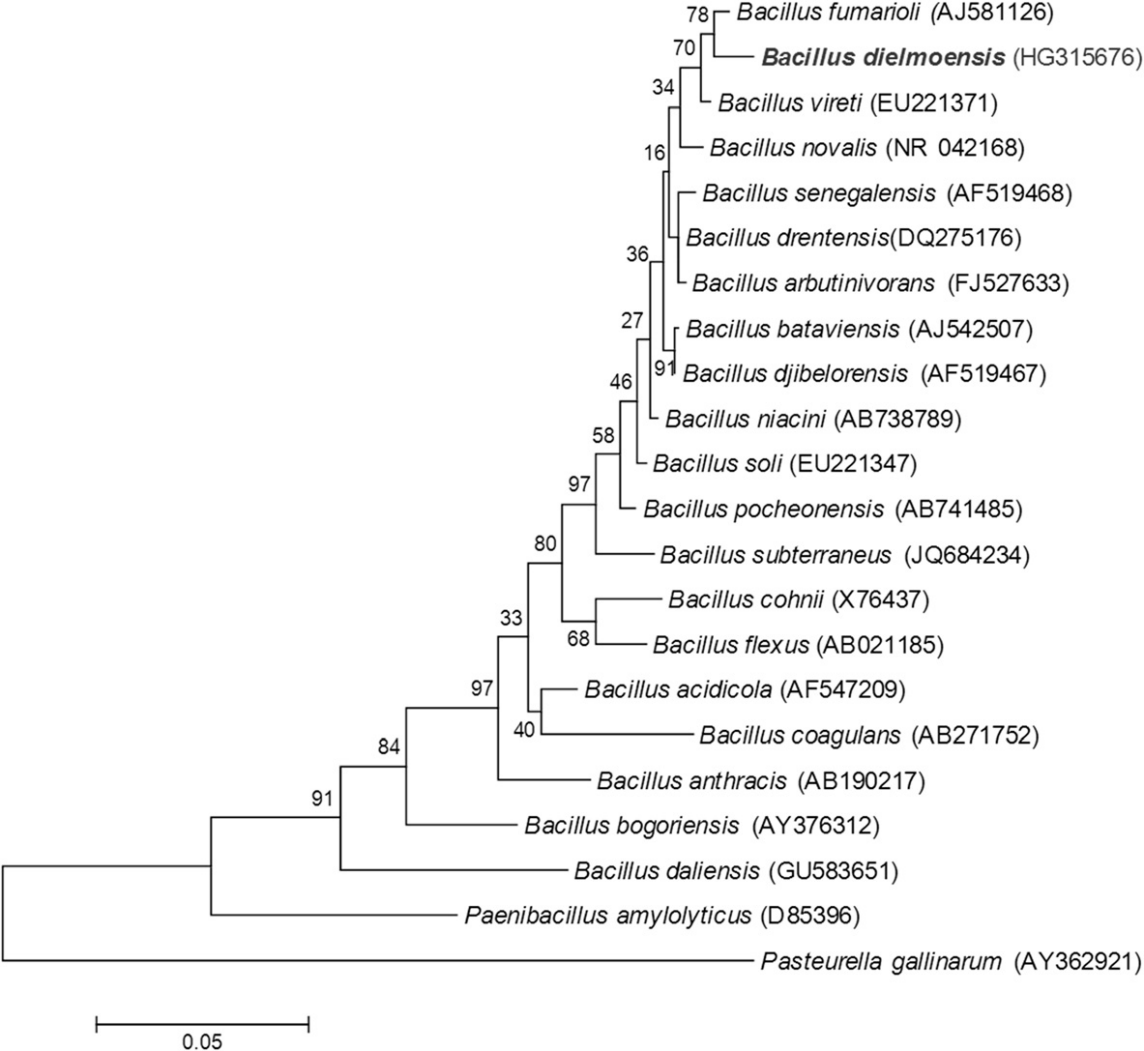
Phylogenetic tree highlighting the position of *Bacillus dielmoensis* strain FF4^T^ relative to the most closely related type strains within the genus *Bacillus*. The strains and their corresponding GenBank accession numbers for 16S rRNA genes are (type = ^T^) and in parenthesis, we indicated GA if the genome is available or GNA if the genome is not available in NCBI web site: *Bacillus fumarioli* strain CIP 106910^T^ (GNA), *B*. *dielmoensis* strain FF4^T^, *B*. *vireti* LMG 21834 (GA : ALAN00000000), *B*. *novalis* strain IDA3307 (GNA), *B*. *senegalensis* strain RS8 (GNA), *B*. *drentensis* strain WN575 (GNA), *B*. *arbutinivorans* strain rif200874 (GNA), *B. bataviensis* strain LMG 21833 (GA: AJLS00000000), *B. djibelorensis* strain RS7 (GNA), *B. niacini* strain NBRC *15566* ^*T*^ (GNA)*, B. soli* strain NBRC 102451^T^, *B. pocheonensis* strain GMC125 (GNA), *B. subterraneus* strain HWG-A11 (GNA), *B. cohnii* strain DSM 6307^T^ (GNA), *B. flexus* strain DSM 1320^T^ (GNA), *B. acidicola* strain 105-2^T^ (GNA), *B. coagulans* strain 2–6 (GA: CP002472), *B. anthracis* (GA: CP008854), *B. bogoriensis* strain ATCC BAA-922 (GA: JHYI00000000), *B. daliensis* strain DLS13 (GNA), *Paenibacillus amylolyticus* strain ATCC 9995^T^ and *Avibacterium gallinarum* strain NCTC 11188^T^. Sequences were aligned using MUSCLE [41], and phylogenetic tree inferred using the Maximum Likelihood method with Kimura 2-parameter model from MEGA software. Numbers at the nodes are percentages of bootstrap values obtained by repeating the analysis 1000 times to generate a majority consensus tree. The scale bar represents a rate of substitution per site of 0.05 was used as outgroup

**Fig. 2:**
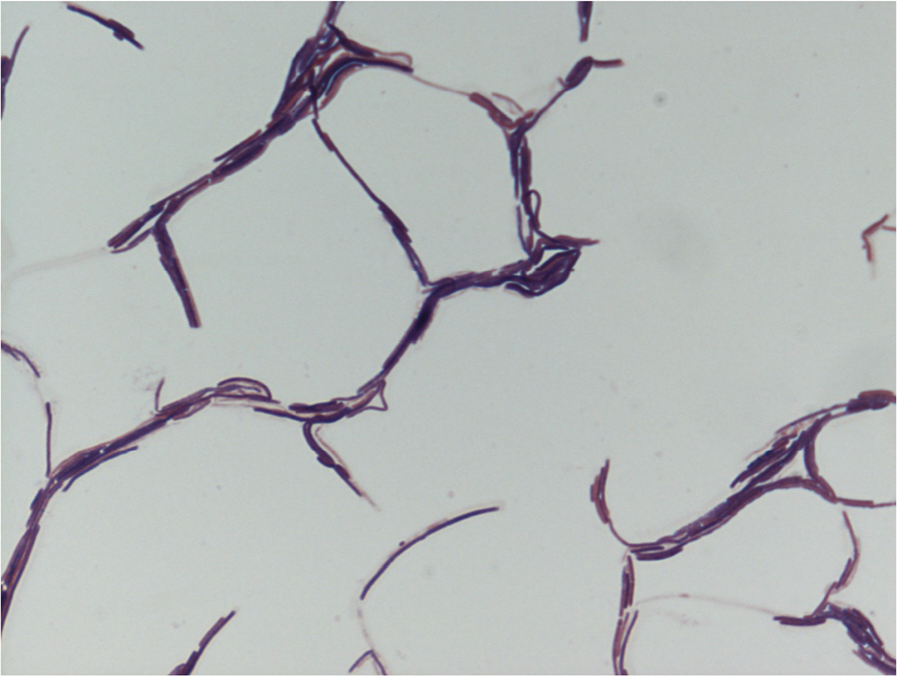
Gram staining of *Bacillus dielmoensis* strain FF4^T^

**Fig. 3:**
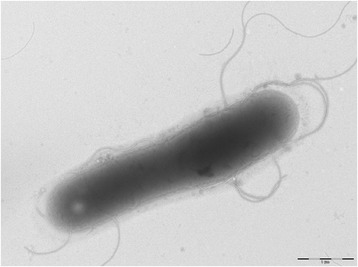
Transmission electron microscopy of *Bacillus dielmoensis* strain FF4^T^, using a Morgani 268D (Philips) at an operating voltage of 60 kV. The scale bar represents 1 μm

A skin sample was collected with a swab from a healthy Senegalese volunteer living in Dielmo (a rural village in the Guinean-Sudanian area in Senegal) in December 2012 (Table 1). This 16-year-old healthy Senegalese female was included in a research project that was approved by the Ministry of Health of Senegal, the assembled village population and the National Ethics Committee of Senegal (CNERS, agreement numbers 09–022), as published elsewhere [13]. The strain FF4^T^ (Table 1) was isolated in December 2012 by cultivation on 5 % sheep blood enriched Columbia agar (BioMérieux, Marcy l’Etoile, France), under aerobic conditions. When the 16S rRNA of *B. dielmoensis* was compared to those of all species with validly published names listed in the list of prokaryotic names with standing in nomenclature from which we also retrieved the 16S rRNA sequences, *B. dielmoensis* strain FF4^T^ exhibited a 97.5 % nucleotide sequence similarity with *B. fumarioli* [14], which is the phylogenetically closest *Bacillus* species (Fig. 1). These values were lower than the 98.7 % 16S rRNA gene sequence threshold recommended by Meier-Kolthoff et al., 2013 to delineate a new species within genus *Bacillus* without carrying out DNA-DNA hybridization [15]. Different growth temperatures (25, 30, 37, 45 °C) were tested. Growth was observed at 30, 37, and 45 °C with the optimal growth obtained at 37 °C after 24 h of incubation. Colonies were 2 mm in diameter and white in color on blood-enriched Colombia agar. Growth of the strain was also tested under anaerobic and microaerophilic conditions using GENbag anaer and GENbag microaer systems, respectively (BioMérieux), and under aerobic conditions, with or without 5 % CO_2_. Growth was observed in all the above mentioned conditions except in anaerobic conditions, where only weak growth was observed. Gram staining showed Gram-positive long rods (Fig. 2). A motility test was also positive. Cells grown on agar have a diameter ranging from 0.5 to 0.8 μm and a length ranging from 2.6 to 5.8 μm as determined by negative staining transmission electron microscopy (Fig. 3).

**Table 1:** Classification and general features of Bacillus dielmoensis strain FF4^T^ [17]

MIGS ID	Property	Term	Evidence code^a^
	Classification	Domain: *Bacteria*	TAS [31]
		Phylum: *Firmicutes*	TAS [32, 33]
		Class: *Firmibacteria*	TAS [34, 35]
		Order: *Bacillales*	TAS [31, 36]
		Family: *Bacillaceae*	TAS [31, 37]
		Genus: *Bacillus*	TAS [31, 38, 39]
		Species: *Bacillus dielmoensis*	IDA
		Type strain: FF4^T^	IDA
	Gram stain	Positive	IDA
	Cell shape	Rod	IDA
	Motility	Motile	IDA
	Sporulation	Non sporulating	IDA
	Temperature range	Mesophile	IDA
	Optimum temperature	37 °C	IDA
	pH range; Optimum	7-7.6; 7.3	IDA
	Carbon source	Unknown	
MIGS-6	Habitat	Human skin	IDA
MIGS-6.3	Salinity	Not growth in BHI medium + 5 % NaCl	IDA
MIGS-22	Oxygen requirement	Aerobic	IDA
MIGS-15	Biotic relationship	Free living	IDA
MIGS-14	Pathogenicity	Unknown	
MIGS-4	Geographic location	Senegal	IDA
MIGS-5	Sample collection time	December 2012	IDA
MIGS-4.1	Latitude	13.7167	IDA
MIGS-4.2	Longitude	– 16.4167	IDA
MIGS-4.4	Altitude	45 m above sea level	IDA

^a^Evidence codes - IDA: Inferred from Direct Assay; TAS: Traceable Author Statement (i.e., a direct report exists in the literature); NAS: Non-traceable Author Statement (i.e., not directly observed for the living, isolated sample, but based on a generally accepted property for the species, or anecdotal evidence). These evidence codes are from the Gene Ontology project [40]. If the evidence is IDA, then the property was directly observed for a live isolate by one of the authors or an expert mentioned in the acknowledgements

Strain FF4^T^ exhibited catalase activity but not oxidase activity. Using the API 50 CH strip (BioMérieux), a positive reaction was observed only for esculin ferric citrate; all other reactions were negative including D-glucose, D-mannose, D-cellobiose, D-trehalose, D-raffinose, starch, D-lyxose, D-fucose, D-arabitol and potassium 2-KetoGluconate. Using the API ZYM strip (BioMérieux), positive reactions were obtained for esterase, esterase lipase, alkaline phosphatase, naphthol-AS-BI-phosphohydrolase, acid phosphatase, β-galactosidase, β-glucuronidase, α-glucosidase and β-glucosidase. No reaction was observed for α-galactosidase, α-chymotrypsin, trypsin, cystine arylamidase, valine arylamidase, leucine arylamidase, N-acetyl-β-glucosaminidase, α-mannosidase and α-fucosidase. Using the API 20E strip (BioMérieux), all the reactions were negative. *B. dielmoensis* is susceptible to amoxicillin, amoxicillin-clavulanic acid, ceftriaxone, imipenem, ciprofloxacin, gentamicin, doxycycline, rifampicin, erythromycin and vancomycin, but resistant to penicillin, trimethoprim-sulfamethoxazole and metronidazole. When compared with representative species from the genus *Bacillus*, *B. dielmoensis* strain FF4^T^ exhibited the phenotypic differences detailed in Additional file 1: Table S1.

**Fig. 4:**
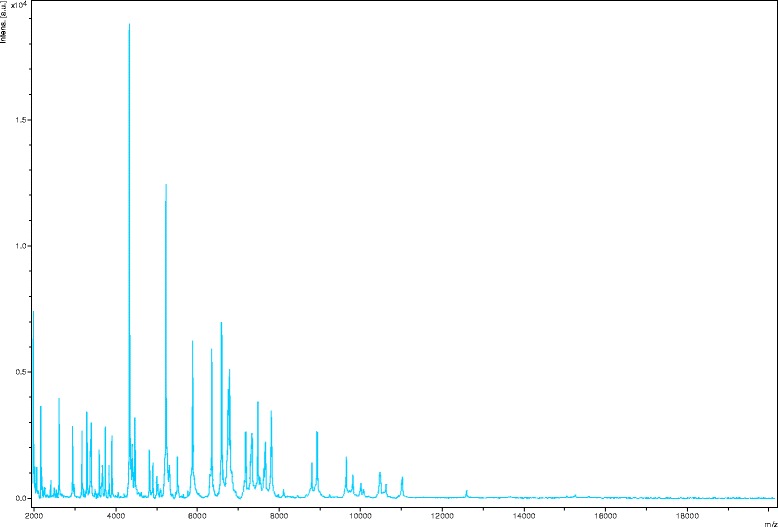
Reference mass spectrum from *Bacillus dielmoensis* strain FF4^T^. Spectra from 12 individual colonies were compared and a reference spectrum was generated

**Fig. 5:**
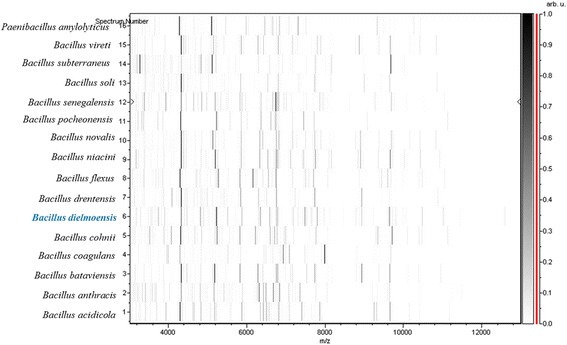
Gel view comparing *Bacillus dielmoensis* strain FF4^T^ to other members of the genus *Bacillus*. The gel view displays the raw spectra of all loaded spectrum files arranged in a pseudo-gel like look. The x-axis records the m/z value. The left y-axis displays the running spectrum number originating from subsequent spectra loading. The peak intensity is expressed by a Gray scale scheme code. The color bar and the right y-axis indicate the relation between the color a peak is displayed with and the peak intensity in arbitrary units. Displayed species are indicated on the left

Matrix-assisted laser-desorption/ionization time-of-flight (MALDI-TOF) MS protein analysis was performed using a Microflex spectrometer (Bruker Daltonics, Leipzig, Germany), as previously reported [16]. The scores previously established by Bruker Daltonics allowing validating or not the identification of species compared to the database of the instrument were applied. Briefly, a score ≥ 2 with a species with a validly published name provided allows the identification at the species level; a score ≥ 1.7 and < 2 allows the identification at the genus level; and a score < 1.7 does not allow any identification. We performed 12 distinct deposits from 12 isolated colonies of strain FF4^T^. Two microliters of matrix solution (saturated solution of alpha-cyano-4-hydroxycinnamic acid) in 50 % acetonitrile and 2.5 % trifluoroacetic-acid were distributed on each smear and submitted at air drying for five minutes. Then, the spectra from the 12 different colonies were imported into the MALDI BioTyper software (version 2.0, Bruker) and analyzed by standard pattern matching (with default parameter settings) against the main spectra of 6,252 bacterial spectra including 199 spectra from 104 *Bacillus* species. Scores ranged from 1.1 to 1.3 were obtained for the strain FF4^T^, suggesting that this isolate was not a member of any known species. The reference mass spectrum from strain FF4^T^ was incremented in our database (Fig. 4). The gel view highlighted spectrum differences with other *Bacillus* species (Fig. 5).

## Genome sequencing information

### Genome project history

The organism was selected for sequencing on the basis of its 16S rRNA similarity, phylogenetic position and phenotypic differences with other members of the genus *Bacillus*, which support that *Bacillus**dielmoensis* strain FF4^T^ likely represents a new bacterial species. Besides, this strain is part of a study aiming to characterize the skin flora of healthy Senegalese people. Currently, there are more of 270 sequenced genomes of *Bacillus* species [2]. The strain FF4^T^ is the first genome of *B. dielmoensis* sp. nov. GenBank accession number is CCAD000000000. It consists of 75 contigs. Table 2 shows the project information and its association with MIGS version 2.0 compliance [17]. Associated MIGS records are detailed in Additional file 2: Table S2.

**Table 2:** Project information

MIGS ID	Property	Term
MIGS-31	Finishing quality	High-quality draft
MIGS-28	Libraries used	One 454 paired-end 3-kb library
MIGS-29	Sequencing platforms	454 GS FLX Titanium
MIGS-31.2	Fold coverage	61x
MIGS-30	Assemblers	Newbler version 2.5.3
MIGS-32	Gene calling method	Prodigal
	Locus Tag	Not determinated
	GenBank ID	CCAD000000000
	GenBank Date of Release	March 12, 2014
	GOLD ID	Gp0101145
	BIOPROJECT	PRJEB4276
MIG-13	Source Material Identifier	DSM 27844
	Project relevance	Study of human skin flora

### Growth conditions and genomic DNA preparation

*Bacillus**dielmoensis* strain FF4^T^ (= CSUR P3026 = DSM 27844) was grown aerobically on 5 % sheep blood-enriched Columbia agar (BioMérieux) at 37 °C. Bacteria growing in four Petri dishes were suspended in 5x100 μL of TE buffer. Then, 150 μL of this suspension were diluted in 350 μL TE buffer 10X, 25 μL proteinase K and 50 μL sodium dodecyl sulfate (SDS) for lysis treatment. This preparation was incubated overnight at 56 °C. DNA was washed 3 times using UltraPure™ Phenol:Chloroform:Isoamyl Alcohol (25:24:1, v/v) (Thermo Fisher Scientific Inc, Waltham, USA) and was precipitated with ethanol at −20 °C during overnight. Following centrifugation, DNA was suspended in 65 μL EB buffer. The genomic DNA concentration was measured at 43.96 ng/μL using the Qubit assay with the high sensitivity kit (Life technologies, Carlsbad, CA, USA).

### Genome sequencing and assembly

Genomic DNA of *Bacillus**dielmoensis* was sequenced on the MiSeq Technology (Illumina Inc, San Diego, CA, USA) with the 2 applications: paired-end and mate-pair. The paired-end and the mate-pair strategies were barcoded in order to be mixed respectively with 10 others genomic projects prepared with the Nextera XT DNA sample prep kit (Illumina) and 11 others projects with the Nextera Mate-Pair sample prep kit (Illumina).

Genomic DNA was diluted to 1ng/μL to prepare the paired-end library. The “tagmentation” step fragmented and tagged the DNA with an optimal size distribution at 1.6 kb. Then, limited cycle PCR amplification (12 cycles) completed the tag adapters and introduced dual-index barcodes. After purification on AMPure XP beads (Beckman Coulter Inc, Fullerton, CA, USA), the libraries were then normalized on specific beads according to the Nextera XT protocol (Illumina). Normalized libraries were pooled into a single library for sequencing on the MiSeq. The pooled single strand library was loaded onto the reagent cartridge and then onto the instrument along with the flow cell. Automated cluster generation and paired-end sequencing with dual index reads were performed in single 39-h run in 2 × 250-bp.

A total of 3.89 Gb sequence was obtained from a 416 K/mm2 cluster density with a cluster passing quality control filters of 95.4 % (7,899,000 clusters). *B. dielmoensis* strain FF4^T^ showed an index representation of 4.95 % within the run and presented 373,015 reads filtered according to the read qualities.

The mate-pair library was prepared with 1 μg of genomic DNA using the Nextera mate-pair Illumina guide. The genomic DNA sample was simultaneously fragmented and tagged with a mate-pair junction adapter. The profile of the fragmentation was validated on an Agilent 2100 BioAnalyzer (Agilent Technologies Inc, Santa Clara, CA, USA) with a DNA 7500 labchip. The DNA fragments ranged in size from 1.5 kb up to 10 kb with an optimal size at 5 kb. No size selection was performed and 600 ng of tagmented fragments were circularized. The circularized DNA was mechanically sheared to small fragments on the Covaris device S2 in microtubes (Covaris, Woburn, MA, USA). The library profile was visualized on a High Sensitivity Bioanalyzer LabChip (Agilent Technologies Inc, Santa Clara, CA, USA) at 586 bp. The libraries were normalized at 2 nM and pooled. After a denaturation step and dilution at 10 pM, the pool of libraries was loaded onto the reagent cartridge and then onto the instrument along with the flow cell. Automated cluster generation and sequencing run were performed in a single 39-h run in a 2×250-bp.

Global information of 3.2 Gb was obtained from a 690 K/mm2 cluster density with a cluster passing quality control filters of 95.4 % (13,264,000 clusters). *B. dielmoensis* strain FF4^T^ shown an index representation of 8.02 % within the run and presented 1,014,931 reads filtered according to the read qualities.

### Genome annotation

Open Reading Frame prediction of the *B. dielmoensis* FF4^T^ genome was performed using Prodigal [18] with default parameters. We excluded the predicted ORFs if they spanned a sequencing gap region. Functional assessment of protein sequences was carried out by comparing them with sequences in the GenBank [19] and Clusters of Orthologous Groups (COG) databases using BLASTP. The tRNAs, rRNAs, signal peptides and transmembrane helices were identified using tRNAscan-SE 1.21 [20], RNAmmer [21], SignalP [22] and TMHMM [23], respectively. Artemis [24] was used for data management whereas DNA Plotter [25] was used for visualization of genomic features. In house perl and bash scripts were used to automate these routine tasks. ORFans were sequences which have no homology in a given database *i.e.* in a non-redundant (nr) or identified if their BLASTP E-value was lower than 1e-03 for alignment lengths greater than 80 amino acids. If alignment lengths were smaller than 80 amino acids, we used an E-value of 1e-05. PHAST was used to identify, annotate and graphically display prophage sequences within bacterial genomes or plasmids [26].

**Fig. 6:**
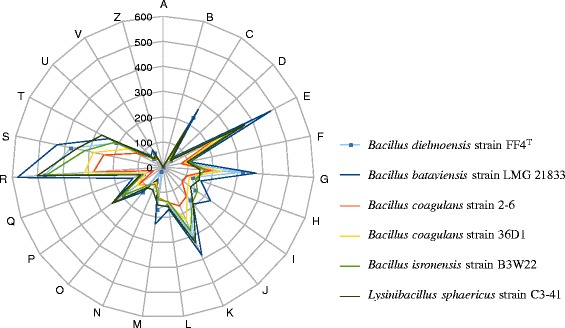
Distribution of functional classes of predicted genes of *B. dielmoensis* strain FF4^T^ along with other *Bacillus* genomes according to the clusters of orthologous groups of proteins

To estimate the nucleotide sequence similarity at the genome level between *B. dielmoensis* and other members of the genus *Bacillus* (Table 3, Fig. 6), orthologous proteins were detected using the Proteinortho software [27] (with the parameters: e-value 1e-5, 30 % percentage of identity, 50 % coverage and algebraic connectivity of 50 %) and genomes compared two by two. After fetching the corresponding nucleotide sequences of orthologous proteins for each pair of genomes, we determined the mean percentage of nucleotide sequence identity using the Needleman-Wunsch global alignment algorithm. The script was created to calculate the average genomic identity of orthologous gene sequences (AGIOS) between genomes using the MAGi software (Marseille Average genomic identity). The script created to calculate AGIOS values was named MAGi (Marseille Average genomic identity) and is written in perl and bioperl modules. GGDC analysis was also performed using the GGDC web server as previously reported [28, 29].

**Table 3:** Orthologous gene comparison of Bacillus dielmoensis strain FF4^T^ with other closely related species. Bold numbers indicate the number of genes from each genome

	*Bacillus dielmoensis* strain FF4^T^	*Bacillus bataviensis* strain LMG 21833	*Bacillus coagulans* strain 2-6	*Bacillus coagulans* strain 36D1	*Bacillus isronensis* strain B3W22	*Lysinibacillus sphaericus* strain C3-41
*Bacillus dielmoensis* strain FF4^T^	**4,308**					
*Bacillus bataviensis* strain LMG 21833	1,888	**5,207**				
*Bacillus coagulans* 2-6	1,517	1,617	**2,971**			
*Bacillus coagulans* strain 36D1	1,631	1,737	1,824	**3,289**		
*Bacillus isronensis* strain B3W22	1,545	1,681	1,332	1,434	**3,883**	
*Lysinibacillus sphaericus* strain C3-41	1,512	1,669	1,321	1,413	1,965	**4,584**

## Genome properties

**Fig. 7:**
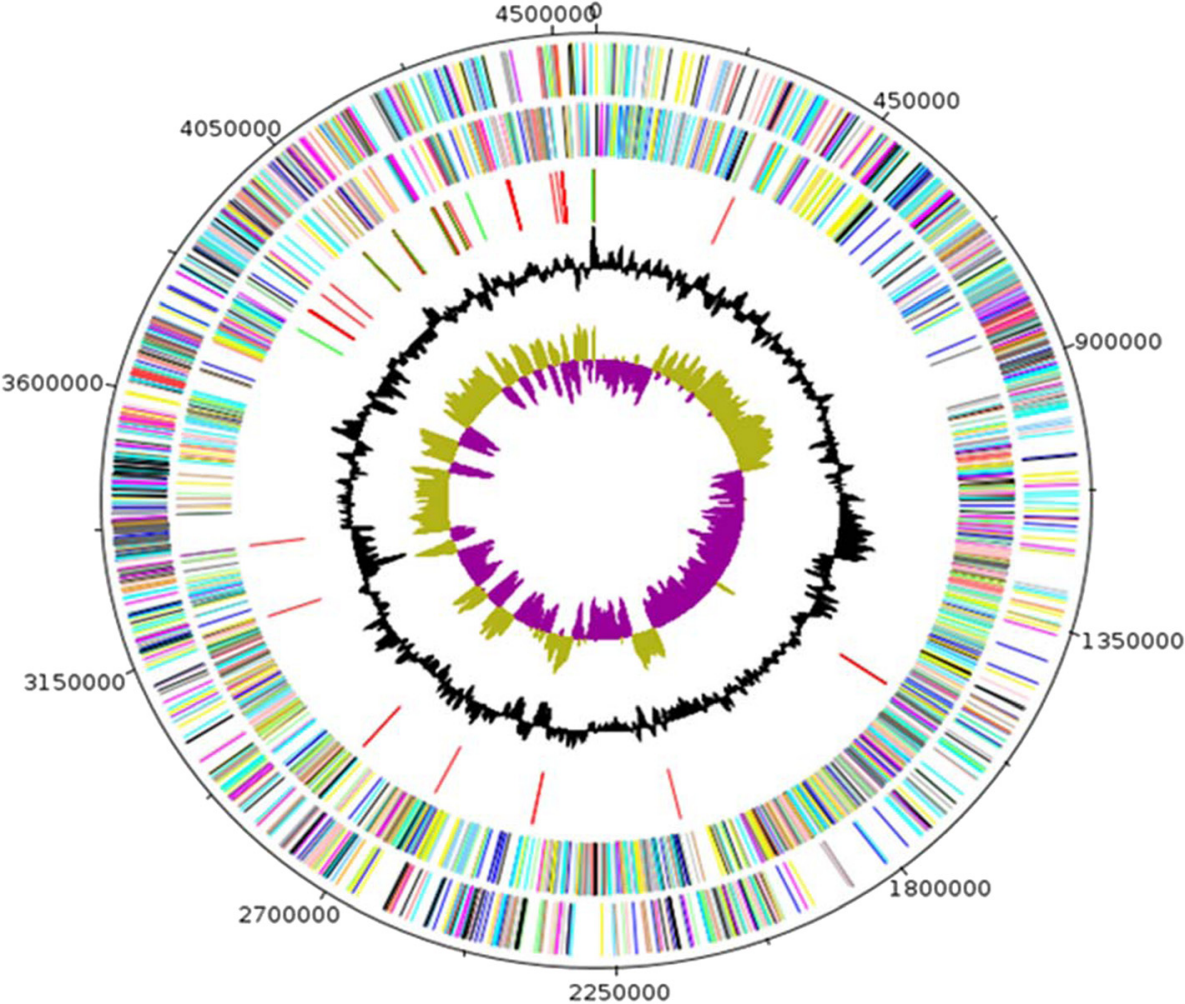
Graphical circular map of the *Bacillus dielmoensis* strain FF4^T^ chromosome. From the outside in, the outer two circles shows open reading frames oriented in the forward (colored by COG categories) and reverse (colored by COG categories) direction, respectively. The third circle marks the rRNA gene operon (red) and tRNA genes (green). The fourth circle shows the G + C% content plot. The inner-most circle shows GC skew, purple indicating negative values whereas olive for positive values

The genome of *B. dielmoensis* strain FF4^T^ is 4,563,381 bp long (1 chromosome but no plasmid) with a 40.8 % G + C content (Fig. 7). Of the 4,465 predicted genes, 4,308 were protein-coding genes and 157 were RNAs. A total of 3,216 genes (74.6 %) were assigned to COGs. A total of 137 genes were annotated as genes with peptide signals. The properties and the statistics of the genome are presented in Table 4. The distribution of genes into COGs functional categories is presented in Table 5.

**Table 4:** Nucleotide content and gene count levels of the genome

Attribute		Genome (total)
	Value	% of total^a^
Genome size (bp)	4,563,381	100
DNA coding (bp)	3,902,509	85.5
DNA G + C (bp)	1,864,870	40.8
DNA scaffolds	nd^b^	
Total genes	4,465	100
Protein-coding genes	4,308	96.4
RNA genes	157	
Pseudo genes	51	1.18
Genes in internal clusters	208	4.82
Genes with function prediction	2,847	66.0
Genes assigned to COGs	3,216	74.6
Genes with Pfam domains	3,235	75.4
Genes with peptide signals	137	3.18
Genes with transmembrane helices	1,189	27.5
CRISPR repeats	3	

^a^The total is based on either the size of genome in base pairs or the total number of protein coding genes in the annotated genome

^b^nd: not determined

**Table 5:** Number of genes associated with the 25 general COG functional categories

Code	Value	% of total^a^	Description
J	155	3.60	Translation
A	0	0.00	RNA processing and modification
K	216	5.01	Transcription
L	126	2.92	Replication, recombination and repair
B	0	0.00	Chromatin structure and dynamics
D	33	0.77	Cell cycle control, mitosis and meiosis
Y	0	0.00	Nuclear structure
V	70	1.62	Defense mechanisms
T	125	2.90	Signal transduction mechanisms
M	152	3.52	Cell wall/membrane biogenesis
N	0	0.00	Cell motility
Z	0	0.00	Cytoskeleton
W	0	0.00	Extracellular structures
U	24	0.55	Intracellular trafficking and secretion
O	98	2.27	Posttranslational modification, protein turnover, chaperones
C	197	4.57	Energy production and conversion
G	233	5.40	Carbohydrate transport and metabolism
E	260	6.03	Amino acid transport and metabolism
F	70	1.62	Nucleotide transport and metabolism
H	86	1.99	Coenzyme transport and metabolism
I	100	2.32	Lipid transport and metabolism
P	147	3.41	Inorganic ion transport and metabolism
Q	26	0.60	Secondary metabolites biosynthesis, transport and catabolism
R	381	8.84	General function prediction only
S	348	8.91	Function unknown
-	369	8.56	Not in COGs

^a^The total is based on the total number of protein coding genes in the annotated genome

## Insights from the genome sequence

Today there are more than 277 sequenced genomes of *Bacillus* species (finished and draft) available in genomic databases [8]. Here, we have compared *B. dielmoensis* genome sequences against other members of genus *Bacillus* including *B. coagulans* strain 2–6, *B. coagulans* strain 36D1, *B. bataviensis* strain LMG 21833, *B. isronensis* strain B3W22, and *Lysinibacillus sphaericus* strain C3-41. The Table 6 shows a comparison of genome size, G + C% content, and number of proteins for selected *Bacillus* genomes for taxonogenomic study.

**Table 6:** Comparison of Bacillus dielmoensis strain FF4^T^ with genomes of other Bacillus species and those of Lysinibacillus sphaericus

Microrganisms	Accession number	Number of proteins	G + C%	Genome size (Mb)
*Bacillus dielmoensis* strain FF4^T^	CCAD000000000	4,308	40.8	4,56
*Bacillus bataviensis* LMG 21833	AJLS00000000	5,207	39.6	5,37
*Bacillus coagulans* 2-6	NC_015634	2,971	47.3	3,07
*Bacillus coagulans* 36D1	NC_016023	3,289	46.5	3,55
*Bacillus isronensis* B3W22	AMCK00000000	3,883	38.8	4,02
*Lysinibacillus sphaericus* C3-41	NC_010382	4,584	37.1	4,82

*Bacillus**dielmoensis* strain FF4^T^ has a G + C content (40.8) lower than those of *Bacillus coagulans* 2*–*6 and 36D1 (47.3 and 46.5, respectively) but higher than those of *B. bataviensis*LMG 21833, *B. isronensis* B3W22 and *L. sphaericus* C3-41 (39.6, 38.8 and 37.1, respectively). As it has been suggested in the literature that the G + C content deviation is at most 1 % within species, these data are an additional argument for the creation of a new taxon [30].

Fig. 6 shows the comparison of gene distribution into COG categories of *B. dielmoensis* with other finished genomes mentioned above. Table 3 presents the numbers of orthologous genes between genome pairs. Table 7 summarizes the AGIOS and dDDH values between the studied genomes. The AGIOS values ranged from 63.25 to 73.22 % at the interspecies level, between *B. dielmoensis* and other species, but was of 95.94 % at the intraspecies level, between the two *B. coagulans* strains. We obtained similar results using the GGDC software, as dDDH values ranged from 0.1057 to 0.2321 between studied species, and was 0.0505 between *B. coagulans* strains. These values confirm the status of *B. dielmoensis* as a new species.

**Table 7:** dDDH values (upper right) and AGIOS values (lower left) obtained by comparison of all studied genomes

	*Bacillus dielmoensis* strain FF4T	*Bacillus bataviensis* strain LMG 21833	*Bacillus coagulans* strain 2-6	*Bacillus coagulans* strain 36D1	*Bacillus isronensis* strain B3W22	*Lysinibacillus sphaericus* strain C3-41
*Bacillus dielmoensis* strain FF4T		0.2321	0.1385	0.1069	0.1866	0.1553
*Bacillus bataviensis* strain LMG 21833	73.22		0.1658	0.1395	0.207	0.1554
*Bacillus coagulans* strain 2-6	64.84	64.71		0.0505	0.1316	0.1057
*Bacillus coagulans* strain 36D1	64.62	64.49	95.94		0.1571	0.107
*Bacillus isronensis* strain B3W22	63.25	63.31	62.21	62.11		0.1981
*Lysinibacillus sphaericus* strain C3-41	63.5	63.61	61.78	61.76	69.18	

## Conclusion

On the basis of phenotypic, phylogenetic and genomic analyses (taxonogenomics), we formally propose the creation of *Bacillus**dielmoensis* sp. nov. that contains the strain FF4^T^ as type strain. The strain was isolated from the skin of a healthy Senegalese 16-year-old female living in Dielmo, Senegal.

## Description of *Bacillus* dielmoensis sp. nov.

*Bacillus**dielmoensis* (di.el.mo.en’sis. L. gen. masc. n. *dielmoensis* of Dielmo, the name of the Senegalese village where the female, from whom strain FF4^T^ was cultivated).

*Bacillus**dielmoensis* is an aerobic Gram-positive bacterium, non-endospore forming and motile. Colonies are 2 mm in diameter and white in color on blood-enriched Colombia agar. Cells are rod-shaped with a mean diameter of 0.6 μm (range 0.5 to 0.8) and a mean length of 4.2 μm (range 2.6 to 5.8). Optimal growth is observed aerobically, weak growth occurs under anaerobic conditions. Growth occurs between 30 and 45 °C, with optimal growth occurring at 37 °C. A catalase activity is present but not oxidase activity. A positive reaction is obtained only for esculin ferric citrate. Positive reactions are observed for esterase, esterase lipase, alkaline phosphatase, naphthol-AS-BI-phosphohydrolase, acid phosphatase, β-galactosidase, β-glucuronidase, α-glucosidase and β-glucosidase. *B. dielmoensis* is susceptible to amoxicillin, amoxicillin-clavulanic acid, ceftriaxone, imipenem, ciprofloxacin, gentamicin, doxycycline, rifampicin, erythromycin and vancomycin, but resistant to penicillin, trimethoprim-sulfamethoxazole and metronidazole.

The G + C content of the genome is 40.8 %. The 16S rRNA and genome sequences are deposited in GenBank under accession numbers HG315676 and CCAD000000000, respectively. The type strain FF4^T^ (= CSUR P3026 = DSM 27844) was isolated from the skin of a healthy female in Dielmo, Senegal.

## Additional files

Additional file 1: Table S1. Differential phenotypic characteristics of *Bacillus dielmoensis* strain FF4^T^ and other *Bacillus* strains [4, 7 14, 42–44]. Additional file 2: Table S2. Associated MIGS record.

## Abbreviations

CSUR: Collection de Souches de l’Unité des Rickettsies
DSM: Deutsche Sammlung von Mikroorganismen
CNERS: National Ethics Committee of Senegal
BHI: Brain Heart Infusion
MALDI-TOF MS: Matrix-assisted laser-desorption/ionization time-of-flight mass spectrometry
TE buffer: Tris-EDTA buffer
SDS: sodium dodecyl sulfate
MAGi: Marseille Average genomic identity
AGIOS: Average Genomic Identity of Orthologous Gene Sequences
GGDC: Genome-to-genome distance calculator
dDDH: Digital DNA-DNA hybridization
