# Supplementary material for: High-quality genome sequence and description of Bacillus dielmoensis strain FF4T sp. nov
Source: Stand Genomic Sci. 2015 Jul 22;10:41. doi: 10.1186/s40793-015-0019-8 (PMC4517664; doi:10.1186/s40793-015-0019-8)
Supplement: Additional file 1: Table S1. — Differential phenotypic characteristics of Bacillus dielmoensis strain FF4T and other Bacillus strains [4, 7 14, 42–44]. [file 40793_2015_19_MOESM1_ESM.docx]

**Additional file 1: Table S1.** Differential phenotypic characteristics of [*Bacillus*](http://dx.doi.org/10.1601/nm.4857) *dielmoensis* strain FF4^T^ and other [*Bacillus*](http://dx.doi.org/10.1601/nm.4857) strains.

| **Properties** | ***B. dielmoensis*** | ***B. fumarioli^a^*** | ***B. vireti^b^*** | ***B. massiliogorillae^c^*** | ***B. berkeleyi*^d^** | ***B. endoradicis*^e^** | ***B. decolorationis*^f^** |
| --- | --- | --- | --- | --- | --- | --- | --- |
| Cell diameter (µm) | 0.5-0.8 | 0.5-0.8 | 0.6-0.9 | 0.8-1.2 | 1.2-1.5 | 0.7-0.9 | 0.5-0.8 |
| Oxygen requirement | Aerobic | Aerobic | Facultative anaerobic | Aerobic | Aerobic | Aerobic | Strictly aerobic |
| Gram stain | + | + | - | Variable | Variable | + | Variable |
| Motility | + | + | + | + | - | + | + |
| Endospore formation | - | + | + | + | + | + | + |
| **Production of** |  |  |  |  |  |  |  |
| Alkaline phosphatase | + | na | na | + | + | na | na |
| Acid phosphatase | + | na | na | + | + | na | na |
| Catalase | + | + | na | + | + | + | + |
| Oxidase | - | na | na | - | + | - | + |
| Nitrate reduction | - | - | + | - | - | + | + |
| Urease | - | - | - | + | - | na | - |
| α-galactosidase | - | na | na | - | - | - | na |
| β-galactosidase | + | na | na | - | - | - | na |
| β-glucuronidase | + | na | na | - | - | na | na |
| α-glucosidase | + | na | na | + | + | - | na |
| β-glucosidase | + | na | na | na | - | - | na |
| Esterase | + | na | na | + | + | na | na |
| Esterase lipase | + | na | na | + | + | na | na |
| naphthol-AS-BI-phosphohydrolase | + | na | na | - | + | na | na |
| N-acetyl-β-glucosaminidase | - | na | na | + | - | na | na |
| α-mannosidase | - | na | na | na | - | na | na |
| α-fucosidase | - | na | na | na | - | na | na |
| **Utilization of** |  |  | na |  |  |  |  |
| 5-keto-gluconate | - | - | - | - | - | na | + |
| D-xylose | - | - | - | - | - | - | - |
| D-fructose | - | + | + | + | - | + | + |
| D-glucose | ~~-~~ | + | + | + | - | + | + |
| D-mannose | ~~-~~ | + | + | + | - | + | + |
| Habitat | human skin | geothermal soils | soil | gorilla gut | sea urchin | soybean | parts of the mural |

^a^[27], ^b^[28] ^c^[4], ^d^[29], ^e^[30], ^f^[31]. na = data not available.
